# Sync or Swim: Navigating the Tides of Menstrual Cycle Messaging on TikTok

**DOI:** 10.1111/psrh.70004

**Published:** 2025-03-17

**Authors:** Emily Pfender, Claire Wanzer, Lotte Mikkers, Amy Bleakley

**Affiliations:** ^1^ Department of OB/GYN University of Pennsylvania Philadelphia Pennsylvania USA; ^2^ Department of Communication University of Delaware Newark Delaware USA; ^3^ Erasmus School of Social and Behavioural Sciences Erasmus University Rotterdam Rotterdam the Netherlands

**Keywords:** cycle syncing, reproductive health, sexual communication, social media

## Abstract

**Objective:**

To understand messaging about cycle syncing on TikTok, a trend that involves harmonizing daily activities and self‐care rituals with various stages of the menstrual cycle.

**Methods:**

We conducted a quantitative content analysis of TikTok videos (*N* = 100). In January 2023, we collected TikTok videos from content creators using the hashtag #cyclesyncing.

**Results:**

About one‐third of creators provided credentials. However, very few mentioned scientific evidence. More than half of the creators recommended aligning diet and exercise with the four menstrual cycle phases. Creators specifically recommended diets and exercises for each menstrual cycle phase.

**Conclusion:**

Cycle syncing content on TikTok oversimplifies a complex literature involving tailoring diet and exercise to the menstrual cycle. Our findings also have implications for previous research pointing to negative discourse about hormonal contraception on social media and problematic messaging about women's reproductive health. More expert voices surrounding women's reproductive health are needed in the evolving social media landscape.

## 1 Introduction

Cycle syncing, a trending phenomenon on TikTok, involves aligning exercise and diet with the four menstrual cycle phases, including the menstrual, follicular, ovulation, and luteal phases [1]. For example, popular press articles suggest that lighter exercise such as yoga or walking is better suited for the menstrual phase [1, 2]. Proponents of cycle syncing believe that the practice is an opportunity to naturally enhance reproductive, physical, and mental well‐being [1]. Although popular, there are some concerns surrounding cycle syncing, including the inability to use hormonal contraceptives if women wish to engage in cycle syncing [3] and the lack of consensus regarding the effect of the four menstrual cycle phases on physical exercise, performance‐related outcomes [4], and diet recommendations [5].^1^

TikTok is a social media platform where users share short‐form videos, often accompanied by music or voice‐overs, with an algorithm‐driven “For You” feed that shows personalized content based on user interactions, making it highly engaging and accessible [6]. TikTok's primary demographic is young people aged 18–34 [6], making it likely that this age group would be exposed to misinformation that circulates on the platform. Considering social media's significant influence on sexual health, it is essential to critically assess the information presented on such platforms. Advocates, wellness coaches, and influencers use social media to share insights on cycle syncing [7] and women's reproductive health [8]. Cycle syncing content is popular on TikTok. The hashtag #cyclesyncing garnered 285 million views, primarily from viewers aged 18–24 [7].

While content creators suggest that tailoring exercise routines to menstrual cycle phases enhances performance, scientific evidence is inconclusive due to methodological flaws [9]. Female athletes report menstrual cycle effects on performance [10], but research yields mixed results [4]. The impact of reproductive hormones on muscle characteristics during different phases remains debated [11]. Similar uncertainty surrounds cycle syncing and diet, with inconsistent findings on energy and nutrient intake during the menstrual cycle [5]. This is criticism around studies linking menstrual cycles and diet because many do not consider an individual's level of daily activity, such as being an athlete [12] or engaging in regular exercise [13]. For example, following cycle syncing principles would prevent a female athlete or daily exerciser from training during certain phases of her cycle, as the menstrual phase recommends less cardio, light weights, and lower impact movements. These principles diverge from training, competitions, and leisure sports, which may inhibit progress toward fitness goals, recreational activities, or professional sports. Additionally, while cycle syncing practices refer to four menstrual cycle phases, scientific research typically only references two [4].

Social media platforms like TikTok can amplify misinformation on cycle syncing. Video content may present misleading or oversimplified information, potentially leading to misconceptions and misguided reproductive health practices [14]. Misinformation includes false claims, exaggerated benefits, and oversimplification of cycle syncing concepts [15]. There is an emerging body of evidence highlighting the spread of sexual health misinformation on TikTok pertaining to sex education and urinary tract infections [16, 17]. For example, Tam and colleagues found that compared to YouTube, TikTok contained more misinformation and provided more inaccurate scientific claims [17]. Medical doctors aim to mitigate sexual health misinformation on TikTok by delivering relatable and authentic sexual health content [18]. However, given the lack of scientific consensus on cycle syncing and the diverse sources and credentials on social media, the legitimacy of cycle syncing sexual health information is questionable. There are concerns about the potential impact of unsupported information on individuals' understanding and implementation of cycle syncing practices. Critical evaluation of cycle syncing content on social media is crucial to navigating the potential pitfalls of misinformation in women's reproductive health.

In this paper, we explore four key questions about cycle syncing content on TikTok. Given the lack of scientific consensus on cycle syncing and the diverse sources and credentials of content creators on social media, the legitimacy of cycle syncing information remains unclear. While some creators may cite scientific research to support their claims, others may rely on anecdotal experiences or generalized wellness trends, raising concerns about the accuracy of the information being disseminated. Another critical consideration is the credibility of those sharing cycle syncing advice. Influencers and wellness coaches often drive health‐related conversations on social media, but their backgrounds and expertise vary widely. Understanding the credentials of content creators discussing cycle syncing can provide insight into the reliability of the information being shared. Beyond questions of credibility, it is also important to analyze how cycle syncing is framed on TikTok. Cycle syncing recommendations often emphasize modifications to exercise and diet, yet existing scientific literature does not fully support these claims. Examining how cycle syncing advocates describe exercise and nutrition can reveal the specific messages being promoted. Finally, content creators frequently highlight the potential benefits of cycle syncing, positioning it as a tool for improved well‐being and hormonal balance. Investigating the benefits that content creators associate with cycle syncing can help clarify the dominant narratives shaping public perceptions of this trend.

## 2 Methods

We selected TikTok videos for analysis, as TikTok is the largest growing social media platform [19] and contains larger amounts of sexual health information compared to other platforms [17]. We selected videos based on (1) ranking and (2) keywords. In January 2023, EJP searched the hashtag “cycle syncing” on TikTok and pulled the top 100 ranked videos for analysis after creating a new account for a 22‐year‐old user in the United States. We obtained videos using the new account and an incognito browser. Incognito offers the opportunity to observe content with minimal information input into the algorithm. EJP stored the top 100 videos in a Microsoft Excel document during data analysis. TikTok considers the top 100 videos as trending with wide reach, and the algorithm is likely to push them to viewers [7]. We conducted all analyses in STATA 18.0.

## 3 Coding Categories

The researchers designed the codebook through a review of the literature and by watching videos with #cyclesyncing. The videos we reviewed recommended dietary and exercise regimes for the four menstrual cycle phases. We added and/or collapsed some codes during training. The final codebook included two sections: creator information and cycle syncing content.

## 4 Creator and Video Information

### 4.1 Credentials

We coded creator's credentials as present or absent. Coders identified credentials when the creator stated their credentials in their bio, in the contents of the video, or in the caption. Thus, we coded credentials when they were written or spoken. For example, many creators wrote “certified health coach” in their profile bio. EJP created these categories based on the credentials mentioned in their bios. Credentials options included clinician, holistic health coach (including nutrition and wellness coaches as well as hormone or period coaches), and fitness instructor/personal trainer (including those who focused on exercise and selling workout programs); we could select all options that applied.

### 4.2 Scientific Research

We coded scientific research as present or absent. If the creator used written or oral communication to attribute their recommendations and tips to research—such as by stating “studies show”—we coded the specific type of research cited. Options included nonprofit groups, government groups, a specific scholarly article, or a non‐specified study.

## 5 Cycle Syncing Information

### 5.1 Exercise

We coded exercise as present if the creator mentioned that they align their exercise with the four menstrual cycle phases. For example, the creator might say, “I exercise in alignment with my cycle.” If exercising differently during the menstrual cycle phases was not orally stated or written in text, we coded it as absent.

### 5.2 Exercise Phase

If we coded exercise as present, coders examined whether specific exercises were verbally or pictorially (e.g., graphic) recommended during each phase of the menstrual cycle, including luteal, ovulatory, follicular, and menstrual phases. For example, a creator might say, “during the luteal phase, I focus on strength training.” We could select more than one type of exercise since many creators advise incorporating multiple forms of exercise in each phase. For example, a creator might say, “during the menstrual phase, I go for walks or do light yoga.”

### 5.3 Diet

We coded diet as present if the creator mentioned that they align their diet with the four menstrual cycle phases. For example, the creator might say, “I eat in alignment with my cycle.” If creators did not verbally link diet to menstrual cycle phases, we coded this item as absent.

### 5.4 Diet Phase

If we coded diet as present, coders examined whether creators verbally or pictorially recommended specific foods during each phase of the menstrual cycle. We coded foods if creators mentioned them verbally or displayed them in a graphic. For example, a creator might say, “during the luteal phase, women need an increased number of calories,” or “during the menstrual phase, I focus on consuming hot foods like soups and stews.”

### 5.5 Benefits

We coded reasons why cycle syncing is beneficial for health, including decreased inflammation, weight loss, improved sleep, or increased libido. For example, a creator might state, “Cycle syncing has significantly improved my sleep,” to indicate a benefit. If no benefits were verbally mentioned, we coded it as “not specified.”

## 6 Coding Protocol

Two graduate students from the University of Delaware coded the TikTok videos. Coders trained in three separate sessions. First, the graduate students met to discuss the codebook, watch sample videos, and refine coding rules. Next, the coders independently coded 40 videos. The training videos did not appear in the top 100 page on TikTok but had the hashtag #cyclesyncing. Once the coders achieved ≥ 0.70 using Gwet's agreement coefficient on the training sample, they independently coded 30% of the final sample [20]. After reaching reliability on 30% of the final sample, one researcher coded the remaining 70 videos. We achieved at least ≥ 0.82 for all variables in the final dataset.

## 7 Results

The videos received an average of 45,235.50 likes and lasted an average of 1:07 min, ranging from 0:06 to 1:52 min (Median = 0:57). Table 1 contains descriptive statistics for creator characteristics and the creator's perceived benefits of cycle syncing.

**TABLE 1:** Credentials, mentions of scientific research, and perceived benefits of cycle syncing among TikTok content creators who discuss cycle syncing (*N* = 100).

Characteristic	% (*n*)
Credentials	30% (30)
Clinician	13% (4)
Health coach	80% (24)
Fitness instructor	17% (5)
Scientific research (i.e., unspecified study)	4% (4)
Benefits	
Balance hormones	11% (11)
Improve acne	10% (10)
Fewer period symptoms	9% (9)
Improve mental health	8% (8)
Improve energy	8% (8)
Reduce inflammation	7% (7)
Optimize health	6% (6)
Improve fertility	5% (5)
Weight loss	4% (4)
Improve digestion	4% (4)
Improve muscle mass	3% (3)
Increase femininity	3% (3)
Increase productivity	2% (2)
Improve sleep	1% (1)
Improve libido	1% (1)

### 7.1 Creator Credentials

About one third of content creators presented credentials (30%, *n* = 30). Credentials included clinicians (*n* = 4), health coaches (*n* = 24), and fitness instructors (*n* = 2).

### 7.2 Scientific Evidence

Only 4% (*n* = 4) mentioned scientific research. The videos that mentioned scientific research referenced unspecified studies without providing an author, publication title, or year of publication.

### 7.3 Recommendations to Cycle Sync Diet and Exercise

TikTok creators (57%, *n* = 57) recommended cycle syncing exercise to the four phases of the menstrual cycle. They mentioned resting (*n* = 14) only during the menstrual phase. They also recommended walking (*n* = 25) and yoga/stretching (*n* = 35) in all four phases, with both most frequently suggested during the menstrual phase. They did not recommend any other activities for the menstrual phase. They suggested barre/Pilates, weightlifting, and cardio in the follicular, ovulatory, and luteal phases but emphasized each differently. They recommended weightlifting (*n* = 36) most often in the luteal phase (*n* = 14), encouraged cardio (*n* = 50) primarily in the follicular (*n* = 20) and ovulatory phases (*n* = 23), and consistently suggested barre/Pilates (*n* = 23) in the luteal phase (*n* = 16).

TikTok creators (54%, *n* = 54) recommended cycle syncing diets for the four menstrual cycle phases, but they mentioned specific nutrients less frequently than specific exercises. Besides hot and raw foods, they primarily focused on different types of vitamins and minerals. They mostly discussed specific vitamins or minerals in the menstrual and luteal phases, with a few exceptions (raw foods, magnesium, B vitamins).

### 7.4 Cycle Syncing Benefits

Creators mentioned 15 benefits in total, with the most common being balancing hormones (11%, *n* = 11), improving acne (10%, *n* = 10), and fewer menstrual symptoms (9%, *n* = 9). However, 63% did not mention any benefits when discussing cycle syncing. Creators typically noted that cycle syncing is important for rebalancing hormones after use of hormonal contraceptives (Table 2).

**TABLE 2:** Exercise and diet recommendations for each of the four menstrual cycle phases among TikTok content creators who recommend cycle syncing (*N* = 100).

Characteristic	Follicular	Ovulatory	Luteal	Menstrual
Exercise				
Resting	—	—	—	14% (14)
Walking	5% (5)	1% (1)	8% (8)	19% (19)
Yoga/stretching	5% (5)	2% (2)	9% (9)	19% (19)
Barre/Pilates	5% (5)	2% (2)	16% (16)	—
Weightlifting	10% (10)	10% (10)	14% (14)	—
Cardio	20% (20)	23% (23)	7% (7)	—
Diet				
Hot foods	—	—	1% (1)	8% (8)
Raw foods	5% (5)	3% (3)	2% (2)	—
Magnesium	—	1% (1)	6% (6)	4% (4)
Iron	—	—	3% (3)	6% (6)
Zinc	—	—	3% (3)	2% (2)
Omega 3	—	—	2% (2)	4% (4)
B vitamins	1% (1)	3% (3)	—	1% (1)
Fiber	—	1% (1)	5% (5)	3% (3)
Increased calories	—	—	7% (7)	—
Decreased calories	1% (1)	—	—	—

## 8 Discussion

Cycle syncing is an emerging health trend on social media (e.g., TikTok, Instagram) that encourages women to exercise and eat in alignment with their menstrual cycles to achieve optimal health [21]. This study seeks to further understand cycle syncing messaging. Cycle syncing content may not be in alignment with our current scientific understanding of menstrual cycles and may perpetuate problematic messaging about exercise and nutrition.

First, our findings suggest that cycle syncing messaging on TikTok contains what could be considered misinformation about exercise and diet. Specifically, uncredentialed creators oversimplified a complex body of literature and exaggerated health benefits. For example, 57% of creators recommended syncing the menstrual cycle to specific types of exercise. Creators commonly recommend high‐intensity activity during the ovulatory phase and rest or low‐intensity exercise during the menstrual phase. However, a large body of research suggests that the menstrual cycle does not commonly affect aerobic and anaerobic performance [4]. The prevalence of scientifically inaccurate information about women's reproductive health is in line with previous social media studies [15, 16, 17, 22, 23].

Second, our findings suggest that cycle syncing content contains problematic messaging about diet and exercise. Creators who encourage women to eat and exercise in alignment with their menstrual cycles suggest that diet and exercise should look the same for all women. For example, the content in this sample suggested that women should rest during the menstrual phase. Research suggests that exercise and diet should take a personalized approach [24]. Additionally, cycle syncing content may reinforce the idea that the menstrual cycle physiologically limits women. This idea can disempower female athletes, as ascribing to the exercise recommendations of cycle syncing may inhibit their ability to perform their sport during certain times of the month.

Placing these findings in the larger context of reproductive health content on social media, cycle syncing trends in the current study have implications for women's decision‐making around contraceptive choices. Women who engage in cycle syncing cannot use hormonal contraceptives [3], which may affect their ability to get pregnant or avoid pregnancy. Previous research documents a trend toward women discontinuing hormonal contraceptive methods and negatively framing these methods on social media [25]. Other research suggests that peers on social media influence contraceptive choices, highlight issues pertaining to drug safety, and delegitimize reproductive health professionals [26]. Foran refers to this conversation on social media as “hormonophobia” or the excessive fear of hormonal contraception [27]. It is unknown whether cycle syncing social media content is an extension of hormonophobia, but our finding that hormonal balance is a benefit of syncing suggests they may be related. Creators discussed the cycle syncing benefit of hormonal balancing in reference to women being on contraceptives for years and disrupting their bodies with synthetic hormones.

## 9 Future Directions and Limitations

This study has several limitations. Even when employing an incognito browser and conducting searches for specific keywords, the algorithmic curation process remains distinct for each person, primarily influenced by their prior search history. As a result, it is not possible to assert with certainty that all individuals searching for cycle syncing videos will encounter precisely the same content. The content seen in incognito mode might not fully represent the diversity of the user base, as it shows what is popular or trending generally, which may not be representative of the experiences of regular users with established interaction histories. Finally, a content analysis can only tell us what is in the messages, not how they affect attitudes and behaviors. Researchers should investigate the effects of cycle syncing videos on attitudes and behaviors.

## 10 Conclusion

Cycle syncing is a popular trend on TikTok, but scientific evidence supporting its effectiveness remains inconclusive. While influencers and wellness coaches promote cycle syncing to enhance physical performance and well‐being, research on the menstrual cycle's impact on exercise and diet is inconsistent. The spread of misinformation on TikTok raises concerns about the credibility of cycle syncing advice, as creators vary widely in expertise and often rely on anecdotal claims. Given the platform's influence on young audiences, it is essential to critically assess the accuracy of health‐related content. Understanding how TikTok creators frame cycle syncing and the credentials of those sharing information can provide insight into this trend and health content more broadly. Future research should explore how social media influences reproductive health decisions and the extent to which cycle syncing content aligns with scientific findings.

## Conflicts of Interest

The authors declare no conflicts of interest.

## Data Availability

The authors are happy to share data and materials upon request.

## Endnotes

^1^
We acknowledge that not everyone who menstruates identifies as a woman or girl. However, throughout this manuscript, we maintain the terminology from the original source material while using inclusive language where relevant and appropriate.
